# Cardio‐ankle vascular index as a predictor of major adverse cardiovascular events in metabolic syndrome patients

**DOI:** 10.1002/clc.23735

**Published:** 2021-09-29

**Authors:** Thosaphol Limpijankit, Prin Vathesatogkit, Dujrudee Matchariyakul, Teerapat Yingchoncharoen, Sukanya Siriyotha, Ammarin Thakkinstian, Piyamitr Sritara

**Affiliations:** ^1^ Division of Cardiology, Department of Medicine, Faculty of Medicine Ramathibodi Hospital Mahidol University Bangkok Thailand; ^2^ Medical and Health Office Electricity Generating Authority of Thailand Bangkruay Thailand; ^3^ Department of Clinical Epidemiology and Biostatistics, Faculty of Medicine Ramathibodi Hospital Mahidol University Bangkok Thailand

**Keywords:** arterial stiffness, cardio‐ankle vascular index (CAVI), major adverse cardiovascular events, metabolic syndrome

## Abstract

**Background:**

Arterial stiffness, as reflected in the cardio‐ankle vascular index (CAVI), is a risk factor for major adverse cardiovascular events (MACEs).

**Hypothesis:**

Combining CAVI and metabolic syndrome (MetS) may enhance prediction of MACEs in a general adult population.

**Methods:**

A total of 3807 employees of the Electricity Generating Authority of Thailand were enrolled in a longitudinal health study during 2007‐2008. Baseline characteristics were collected and CAVI determined. Subjects with previous coronary artery disease or stroke were excluded from analysis. MetS was defined using the modified NCEP‐ATP III for Asians. The primary study endpoint was occurrence of a MACE (myocardial infarction, stroke, or cardiovascular death).

**Results:**

MetS was present in 39.2% at study baseline. The prevalence of CAVI > 9 was higher in subjects with MetS compared to those without (33.7% vs. 28.5%, *P* = 0.001). During the 12.4 ± 0.6 years follow‐up, 227 participants developed MACEs and 350 died. MetS was more common in patients who developed a MACE (8.2% vs. 5.0%, *p* < 0.001) than was non‐MetS, but it was not a significant risk after adjusting covariables. Participants with CAVI > 9 had greater risk for MACEs 1.34 (95% CI: 1.01, 1.79) relative to those with CAVI < 9. Participants with both MetS and CAVI > 9 had the worst outcomes, with the highest frequency of MACEs, among the four groups.

**Conclusion:**

Arterial stiffness assessed by CAVI may enhance prediction of future MACEs, adding to the null predictive power of MetS. This index can be used to motivate MetS patients to modify their life‐styles for prevention.

## 1 INTRODUCTION

Metabolic syndrome (MetS) is associated with an increase in major adverse cardiovascular events (MACEs) and all‐cause death (ACD).^1^, ^2^ Patients with MetS have a 2‐fold increase in cardiovascular events (CVEs) and a 1.5‐fold increase in ACD.^3^ The pathogenesis of atherosclerosis in MetS is related to uncontrolled risk factors, chronic inflammatory processes, and a prothrombotic state. These mechanisms cause progression of premature atherosclerosis and development of early onset type 2 diabetes mellitus (DM).

The prevalence of MetS in adult populations is increasing worldwide as well as in Asia, from 10%–15% in the last decade to 20%–25% currently.^4^, ^5^, ^6^ To prevent CVEs and to reduce death, it is important to encourage these vulnerable populations to reduce their risk factors. There are many non‐invasive tests available to detect subclinical atherosclerosis in high‐risk patients including cardiac stress test, carotid duplex ultrasound, coronary calcium score, coronary computed tomographic angiography, and so forth. However, all of these tests have limitations as screening tools because of their cost and lack of evidence that their use is associated with a reduction in CVEs and death.^7^, ^8^, ^9^, ^10^ Thus, there remains a need to identify non‐invasive physiologic tests that can be used to risk‐stratify MetS subjects, especially early in their disease process.

Arterial stiffness is one of the earliest properties accompanying the development of atherosclerosis.^11^ Cardio‐ankle vascular index (CAVI), a newly developed indicator of arterial stiffness, has been widely used as a surrogate marker for cardiovascular disease (CVD).^12^, ^13^ This non‐invasive tool might provide earlier diagnosis and identification of subjects at high risk for CVEs and death. Unlike brachial‐ankle pulse wave velocity (PWV), CAVI has the advantage of being unaffected by blood pressure (BP) and gives reproducible results in the clinic. Previous studies have shown that CAVI is able to diagnose subclinical atherosclerosis and to predict future CVEs.^14^, ^15^ In patients with metabolic disorders, CAVI has been reported to be a predictor of CVEs, independent of conventional atherosclerosis risk factors.^16^, ^17^ This study investigated whether combining arterial stiffness (as reflected by the CAVI) and status regarding MetS may further enhance prediction of MACEs in a general adult population.

## 2 METHODS

### 2.1 Study design

The Electricity Generating Authority of Thailand (EGAT) study is a large prospective cohort study conducted in adult employees to better understand the occurrence and influence of risk factors on progression of CVD in Thais.^18^ This longitudinal health study is composed of three cohorts (EGAT 1, 2, and 3; started in 1985, 1998, and 2009, respectively) who were studied at enrolment and followed up every 5 years. The study protocol conforms to the ethical guidelines of the 1975 Declaration of Helsinki and was approved by the Ethics Committee of the Faculty of Medicine, Ramathibodi Hospital, Mahidol University (COA. No. MURA2018/548). All participants provided their informed consent.

### 2.2 Data collection

A total of 3807 subjects from the EGAT 1/3 and EGAT 2/4 cohorts seen at follow up in 2007 and 2008 were enrolled into this study. Characteristics at this 2007/2008 time point, including age, sex, atherosclerosis risk factors (e.g., smoking, DM, hypertension, and dyslipidemia), body mass‐index (BMI, kg/m^2^), waist circumference (WC), and underlying diseases, were noted as ‘baseline’ and recorded. To detect MetS, a newly diagnosed risk factor, fasting blood samples for plasma glucose (FPG) and lipid profile [total cholesterol, high density lipoprotein‐cholesterol (HDL‐C), low density lipoprotein‐cholesterol (LDL‐C), triglyceride (TG)] were collected from these subjects. DM was defined as an overnight fasting plasma glucose (FPG) ≥126 mg/dl or taking anti‐diabetic medications. Hypertension was defined as systolic BP (SBP) ≥140 mm Hg and/or diastolic BP (DBP) ≥90 mm Hg, or taking anti‐hypertensive medication. Dyslipidemia was defined as elevated total cholesterol ≥200 mg/dl or LDL‐C ≥ 130 mg/dl or taking statin medications. For each subject, ankle‐brachial index (ABI) and CAVI were also determined at baseline.

### 2.3 Metabolic syndrome definitions

MetS was defined accordingly to the National Cholesterol Education Program Expert Panel and Adult Treatment Panel III (NCEP‐ATP III) criteria,^1^ that was modified in 2004 and adapted for Asians.^19^ In this study a diagnosis of MetS required the presence of three or more of the following: (1) WC ≥90 cm in men and ≥ 80 cm in women, (2) serum TG ≥150 mg/dl or under treatment, (3) HDL‐C < 40 mg/dl in men and < 50 mg/dl in women, (4) SBP ≥130 or DBP ≥85 mm Hg, or under treatment, (5) FPG ≥100 mg/dl or under treatment. These criteria were used in our study because of their stronger association with CVD and risk of death than the criteria of the International Diabetes Federation and American Heart Association/National Heart Lung Blood Institute.^20^

### 2.4 Measurement of CAVI

**FIGURE 1:**
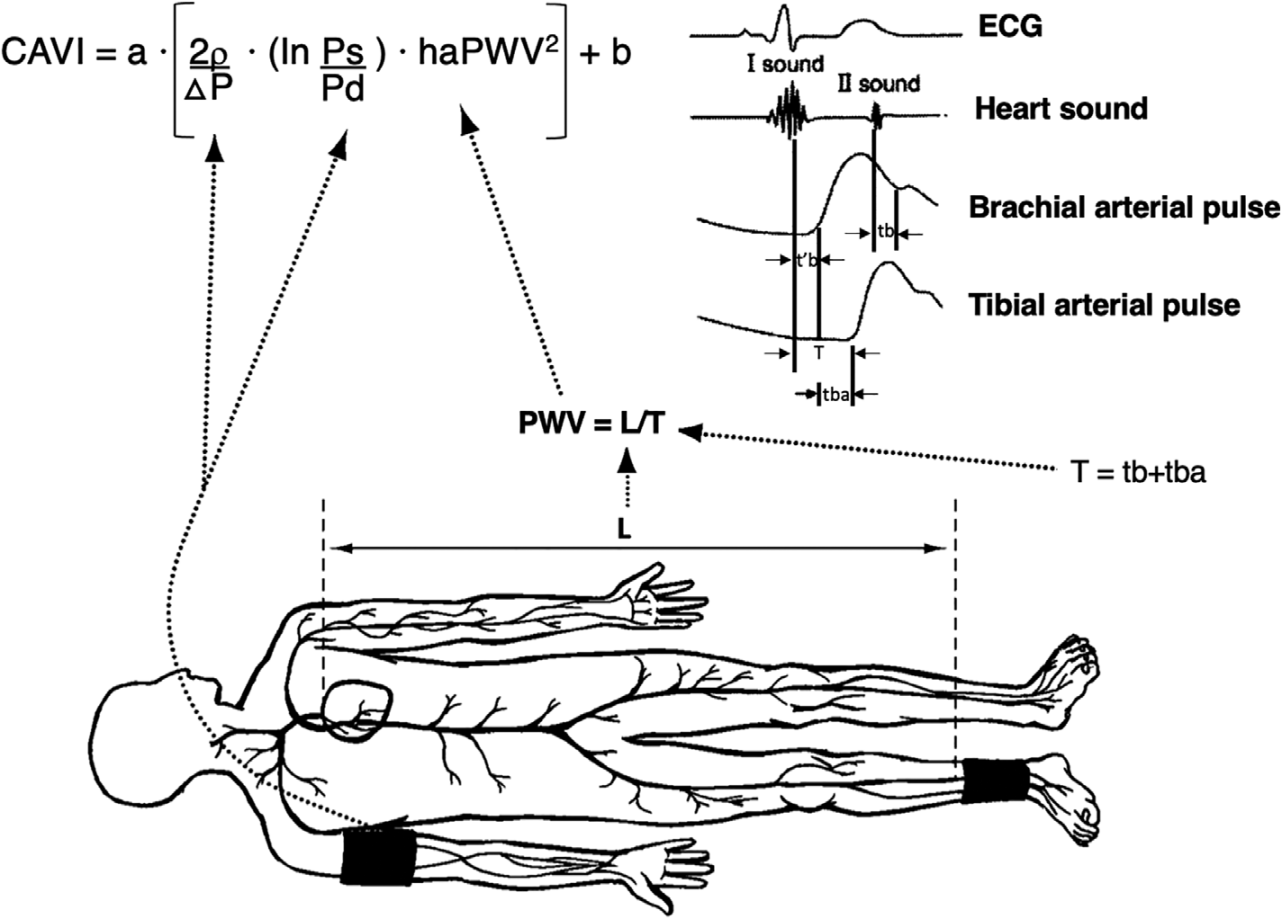
Schematic illustration of cardio‐ ankle vascular index (CAVI) measurement. ECG, electrocardiogram; haPWV, heart ankle pulse wave velocity; Ps, systolic blood pressure; Pd, diastolic blood pressure; DP, Ps–Pd; *ρ*, blood density; tba, time between rise in brachial pulse wave and rise in ankle pulse wave; tb, time between closing sound of aortic valve and notch in brachial pulse wave; t'b, time between opening sound of aortic valve and rise in brachial pulse wave; *a* and *b*, scale conversion constants. *Source*: Modified from Shirai et al.^21^

CAVI was measured using a Vasera VS‐1000 vascular screening system (Fukuda Denshi, Japan).^21^ In brief, patients were in the supine position with pressure cuffs applied bilaterally to upper arms and ankles. After resting for 10 min, the electrocardiogram and phonocardiogram were monitored the heart rhythm and heart sound, respectively. Pulse wave velocity (PWV) was obtained by measuring the distance between the aortic valve to the ankle (L) divided by time for the pulse wave to propagate from the aortic valve to the ankle (T). CAVI is derived from this PWV and BP using the following equation: CAVI = *a*{(2*ρ*/Δ*P*) × ln(Ps/Pd) × haPWV^2^} + *b*, where Ps and Pd are the SBP and DBP, respectively; PWV is the pulse wave velocity between the heart and ankle; *ρ* is blood density; Δ*P* is Ps–Pd; *a* and *b* are scale conversion constants **(**Figure 1 **)**.^21^ This index is calculated from the heart‐ankle pulse wave velocity (haPWV), and adjusted for BP based on a stiffness parameter. In our study, the mean value of right and left CAVI was used for analyses. According to the manufacturer, values <8 are considered as normal, 8 to <9 as borderline, and ≥ 9 as high and suggestive of the presence of arteriosclerosis.^22^ In our study, we used CAVI values ≥9 as a candidate risk factor for subclinical atherosclerosis in MetS patients.^23^ This machine also measured the ABI, another physiological test for diagnosis of peripheral arterial disease.

### 2.5 Enrolled subjects

Subjects were randomly enrolled from the ongoing EGAT study, among EGAT employees in the Bangkok metropolitan area (EGAT 1) and at three different sites in Western and Northern Thailand (EGAT 2). The age range was selected as 35–54 years old, to maximize the probability of CVD events, given that the retirement age in EGAT in 1985 was 55 years. A large variety of people were recruited, from illiterates working as cleaners to truck drivers, security guards, office clerks, administrators, architectures, engineers, field explorers, lecturers, lawyers, health‐care practitioners, and those on executive boards.

Inclusion criteria included the presence of atherosclerosis risk factors but absence of related symptoms. Exclusion criteria included a diagnosis CVD at baseline, either coronary artery disease (CAD), myocardial infarction (MI), or stroke. To ensure correct CAVI measurements, participants with any of the following characteristics were also excluded: 1) abnormal ABI <0.9, 2) interference waveform of phonocardiogram and arterial (brachial or ankle) pulse waves, 3) atrial fibrillation, and 4) outlier CAVI values (≤3 or ≥ 18).^22^

### 2.6 Follow‐up and clinical outcomes

Following the baseline assessments in 2007–2008, all participants were resurveyed for possible study outcomes in December 2019. They were contacted by telephone and invitation letters. The primary endpoint was occurrence of a MACE (i.e., non‐fatal MI, non‐fatal stroke, or cardiovascular death) and the secondary endpoint was ACD. Cardiovascular death was defined as a CAD death or stroke death. CAD was defined as the presence of at least one of the following criteria: angina, acute coronary syndrome, acute MI, a significant (>70% diameter stenosis) lesion on coronary angiography, revascularization (PCI or CABG), and documented myocardial ischemia during exercise testing. Stroke was defined as a history of ischemic or hemorrhagic stroke, or transient ischemic attack (TIA). If a subject had two cardiovascular events (i.e., MI or stroke), only the first event was counted. If a subject died of a cardiovascular disease, this subject was counted as both a MACE and an ACD. The names of study participants who did not reply to the surveys or who may have died during the follow‐up period, were cross‐checked against death databases maintained by the National Health Security Office (which holds hospital discharge records) and the Department of Provincial Administration of the Ministry of the Interior (which compiles death certificates) to ascertain vital status. The cause of death was assessed by an independent adjudication committee, comprised of cardiologists and neurologists, and classified into CAD (fatal MI or sudden unexplained death), stroke (including subarachnoid hemorrhage), other vascular death (e.g., heart failure, valvular heart disease, or PAD), non‐cardiovascular death (i.e., malignancy, respiratory and gastrointestinal diseases, accident, sepsis, metabolic (DM, hypertension, dyslipidemia), or unknown).

### 2.7 Statistical analysis

Characteristics at enrollment were described using mean ± SD for continuous variables and percentages for categorical variables. These corresponding variables for the MetS groups were compared using a two‐sided Student's *t*–test or Chi‐square test. Kaplan–Meier (KM) curves of MACEs were constructed according to MetS and CAVI groups, and these KM curves were compared using a log‐rank test. Multiple Cox proportional hazard models were used to determine whether MetS and CAVI were associated with MACEs. A multivariate Cox regression model was applied to assess association effects of MetS and CAVI on MACEs. Atherosclerosis risk factors, including age, sex, BMI, smoking, DM, hypertension, and dyslipidemia, were also used to adjust the multivariate Cox models if their *p*‐values in the simple Cox model were less than .20. Forward selection was applied to select only significant risk factors in the MACE model that already contained MetS and CAVI. Hazard ratios (HRs) along with 95% confidence intervals (CIs) were then estimated. A *p*‐value <.05 was considered statistically significant. All statistical analyses were performed using STATA version 16 (Stata Corp., College Station, Texas, USA).

## 3 RESULTS

### 3.1 Baseline characteristics of participants

**FIGURE 2:**
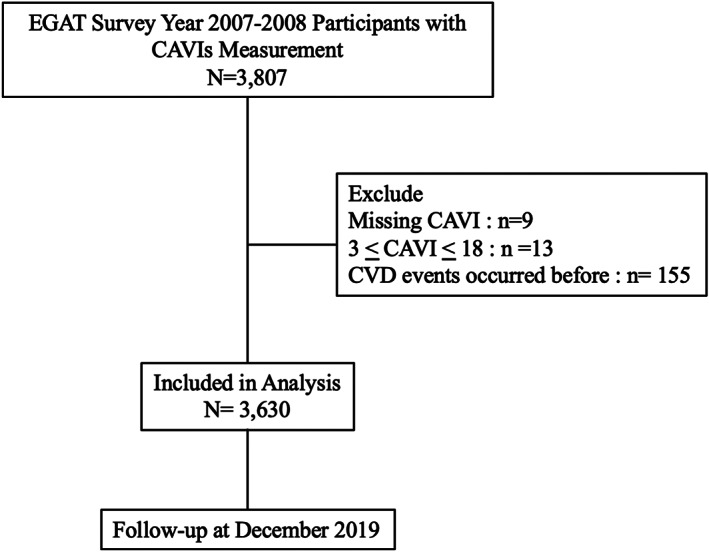
Flow of selection of patients in analysis

Out of 3807 participants, 3630 were included in the analysis (Figure 2); their age, BMI, and percent male were 57.4 ± 7.3 years, 24.6 kg/m^2^, and 73.2%, respectively. The overall prevalence of MetS was 39.2%. Baseline characteristics, classified by MetS groups, were compared and are shown in Table 1. Participants with MetS were older, and more frequently smoked, DM, hypertension, dyslipidemia, and had higher BMI, waist circumference, FPG, TG, and lower total cholesterol, LDL‐C, and HDL‐C. In addition, significant arterial stiffness, defined as CAVI≥9, was found more frequently in individuals with MetS than non‐MetS (33.7% vs 28.5%, respectively: *p* = .001). ABI classified as above or below the median (1.12), was found to be independent of the presence of MetS.

**TABLE 1:** Baseline characteristics of all participants (*N* = 3630)

Variable	Metabolic syndrome		*p*‐value
	Yes	No	
	(*n* = 1423)	(*n* = 2207)	
CAVI, *n* (%)			
≥ 9	480 (33.7)	628 (28.5)	.001
< 9	943 (66.3)	1579 (71.5)	
ABI, *n* (%)			
≥ 1.12	723 (50.8)	1052 (47.7)	.065
< 1.12	700 (49.2)	1155 (52.3)	
Age, years,	58.5 ± 7.3	56.7 ± 7.3	<.001
Gender, *n* (%)			
Male	1066 (74.9)	1592 (72.1)	.065
Female	357 (25.1)	615 (27.9)	
BMI, kg/m^2^,	26.5 ± 3.5	23.4 ± 3.1	<.001
Waist circumference, cm,	93.6 ± 8.3	84.8 ± 8.6	<.001
Smoker, *n* (%)			
Yes	731 (51.7)	1050 (47.8)	.022
No	684 (48.3)	1148 (52.2)	
DM, *n* (%)			
Yes	356 (25.2)	115 (5.3)	<.001
No	1059 (74.8)	2065 (94.7)	
Hypertension, *n* (%)			
Yes	826 (58.8)	552 (25.4)	<.001
No	579 (41.2)	1620 (74.6)	
Dyslipidemia, *n* (%)			
Yes	796 (57.4)	1047 (49.3)	<.001
No	591 (42.6)	1078 (50.7)	
Cholesterol, mg/dl	221.2 ± 45.6	224.7 ± 40.9	.017
LDL‐C,^a^ mg/dl	140.8 ± 40.7	149.0 ± 37.6	<.001
HDL‐C, mg/dl	48.6 ± 13.5	57.6 ± 13.8	<.001
FPG, mg/dl	113.3 ± 33.1	95.8 ± 17.4	<.001
TG, mg/dl, median (IQR)	167 (123, 224)	106 (79, 137)	<.001

Abbreviations: ABI, ankle‐brachial index; BMI, body mass index; CAVI, cardio‐ankle vascular index; DM, diabetes mellitus; FPG, fasting plasma glucose; HDL, high density lipoprotein‐cholesterol; IQR, interquartile range; LDL, low density lipoprotein‐cholesterol; MetS, metabolic syndrome; TG, triglyceride; WC, waist circumference.

a LDL‐C results were confounded by inclusion of subjects on medication.

### 3.2 Clinical outcomes

During the follow‐up (a period which averaged 12.4 ± 0.6 years), 227 participants experienced a MACE and 350 died. Participants with MetS at baseline, compared to those without, developed more MACEs (8.2% vs 5.0%, *p* < .001) and had a higher ACD (11.2% vs 8.7%, *p* = .012). The percentages of both fatal and non‐fatal MI events were significantly higher in the MetS group (1.8% vs 1.0%, *p* = .045, and 3.5% vs 1.9%, *p* < .001, respectively), whereas there were no significant differences in fatal or non‐fatal strokes (0.3% vs 0.2%, *p* = .531, and 1.8% vs 1.3%, *p* = .410, respectively). There was also no significant difference in non‐CVD death between the two groups (7.8% vs 6.7%, *p* = .211); these non‐CVD deaths consisted of malignancy (49.8%), respiratory and gastrointestinal diseases (10.9%), accidents (7.3%), sepsis (11.2%), metabolic (1.8%), and miscellaneous or unknown (18.9%).

### 3.3 Predictors associated with MACEs

Using bivariate analysis, MetS, CAVI, age, sex, ABI, BMI, waist circumference, smoking, FPG, DM, and hypertension were each found to be significantly associated with MACEs, see Table 2. Dyslipidemia and TG had a tentative to be associated with MACEs. After adjustment for confounders (Table 3), factors independently associated with higher risk of future MACEs were CAVI ≥ 9, old age, male sex, BMI, DM, and hypertension with corresponding HRs (95% CI) of 1.34 (1.01, 1.79), 1.03 (1.01, 1.05), 2.66 (1.75, 4.06), 1.04 (1.00, 1.09), 1.76 (1.27, 2.45), and 1.44 (1.06, 1.94), respectively. MetS and dyslipidemia were not significantly associated with higher risk of MACEs, with the HRs of 1.05 (0.76, 1.44) and 1.10 (0.83, 1.46), respectively.

**TABLE 2:** Patient characteristics associated with MACEs

Variable	MACEs		
	Yes	No	
	(*n* = 227)	(*n* = 3403)	*p*‐value
Metabolic syndrome, *n* (%)			
MetS	117 (8.2)	1306 (91.8)	<.001
Non‐MetS	110 (5.0)	2097 (95.0)	
CAVI, *n* (%)			
≥ 9	90 (8.1)	1018 (91.9)	.002
< 9	137 (5.4)	2385 (94.6)	
ABI, *n* (%)			
≥ 1.12	130 (7.3)	1645 (92.7)	.009
< 1.12	97 (5.2)	1758 (94.8)	
Age, years	60.1 ± 7.7	57.2 ± 7.3	<.001
Gender, *n* (%)			
Male	197 (7.4)	2461 (92.6)	<.001
Female	30 (3.1)	942 (96.9)	
BMI, kg/m^2^	25.4 ± 3.2	24.6 ± 3.6	.001
Waist circumference, cm	91.5 ± 9.3	88.0 ± 9.5	<.001
Smoker, *n* (%)			
Yes	134 (7.5)	1647 (92.5)	.001
No	90 (4.9)	1742 (95.1)	
DM, *n* (%)			
Yes	64 (13.6)	407 (86.4)	<.001
No	163 (5.2)	2961 (94.8)	
Hypertension, *n* (%)			
Yes	123 (8.9)	1255 (91.1)	<.001
No	98 (4.5)	2101 (95.5)	
Dyslipidemia, *n* (%)			
Yes	127 (6.9)	1716 (93.1)	.055
No	89 (5.3)	1580 (94.7)	
Cholesterol, mg/dl	222.9 ± 47.9	223.3 ± 42.5	.877
LDL,^a^ mg/dl	145.1 ± 44.0	145.8 ± 38.7	.797
HDL‐C, mg/dl	53.5 ± 14.8	54.1 ± 14.4	.536
FPG, mg/dl	111.6 ± 41.6	102.1 ± 24.7	<.001
TG, mg/dl, median (IQR)	132 (95, 184)	123 (88, 172)	.081

Abbreviations: ABI, ankle‐brachial index; BMI, body mass index; CAVI, cardio‐ankle vascular index; DM, diabetes mellitus; FPG, fasting plasma glucose; HDL, high density lipoprotein‐cholesterol; IQR, interquartile range; LDL, low density lipoprotein‐cholesterol; MetS, metabolic syndrome; TG, triglyceride; WC, waist circumference.

a LDL‐C results were confounded by inclusion of subjects on medication.

**TABLE 3:** Multivariate predictors of MACEs after adjustment for confounders

Variable	MACEs	*p*‐value
	HR (95% CI)	
MetS vs non‐MetS	1.05 (0.76, 1.44)	.779
CAVI ≥9 vs < 9	1.34 (1.01, 1.79)	.048
Age, years	1.03 (1.01, 1.05)	.001
Male vs female	2.66 (1.75, 4.06)	<.001
BMI, kg/m^2^	1.04 (1.00, 1.09)	.041
DM vs none	1.76 (1.27, 2.45)	.001
Hypertension vs none	1.44 (1.06, 1.94)	.018
Dyslipidemia vs none	1.10 (0.83, 1.46)	.508

Abbreviations: ABI, ankle‐brachial index; BMI, body mass index; CAVI, cardio‐ankle vascular index; DM, diabetes mellitus; FPG, fasting plasma glucose; HDL, high density lipoprotein‐cholesterol; IQR, interquartile range; LDL, low density lipoprotein‐cholesterol; MetS, metabolic syndrome; TG, triglyceride; WC, waist circumference.

### 3.4 Interaction effects between MetS and CAVI on MACEs

**FIGURE 3:**
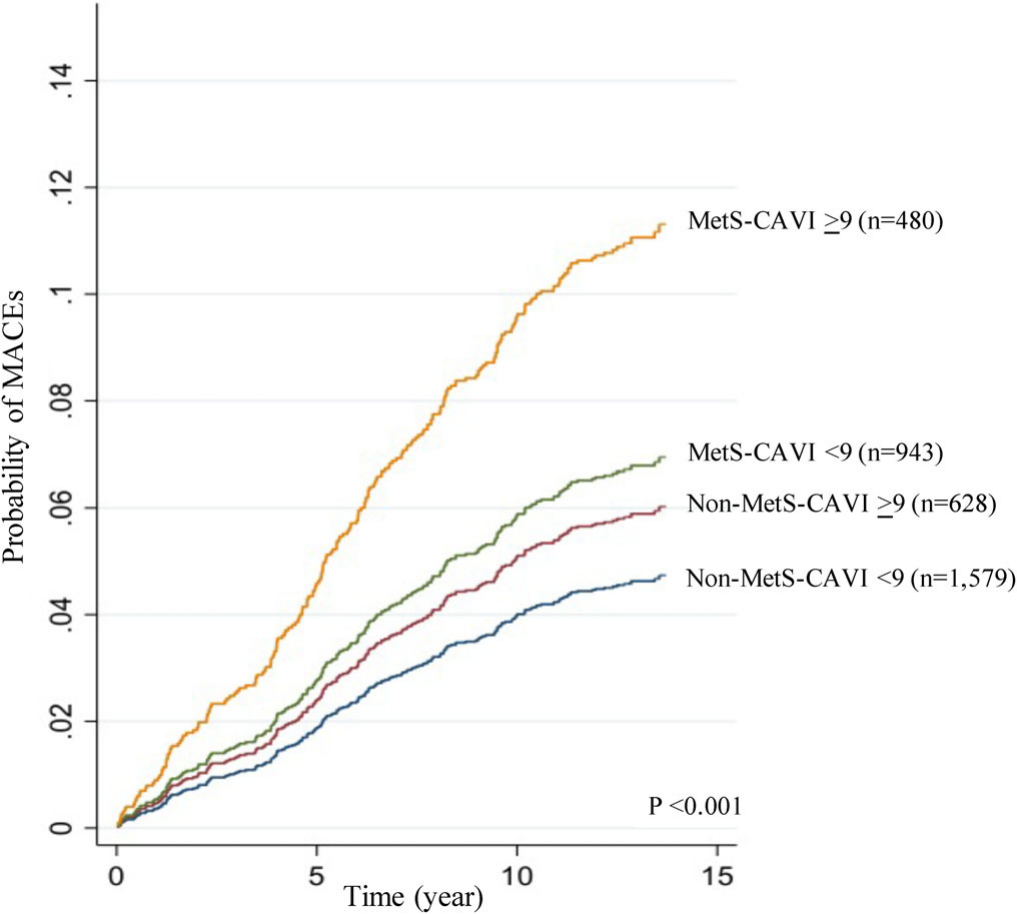
Probability of major adverse cardiovascular events (MACEs) among 3630 subjects grouped by MetS and CAVI status

The interaction effect between MetS and CAVI on MACEs was assessed. Participants with both MetS and CAVI ≥ 9 had the worst outcomes, with the highest frequency of MACEs, among the four groups. Kaplan–Meier curves demonstrate these differences: the highest occurrence of MACEs was in the participants with MetS and CAVI ≥ 9, followed by MetS and CAVI < 9, non‐MetS and CAVI ≥ 9, and non‐MetS and CAVI < 9 (*p* < .001, Figure 3). Participants with MetS and CAVI ≥ 9 had a MACE about 67% higher than the MetS and CAVI < 9 groups and 93% higher than the non‐MetS and CAVI ≥ 9. The differences between groups were visible as early as year 1, and persisted through the follow‐up period.

## 4 DISCUSSION

This is the first study to assess the effects of MetS and CAVI on the occurrence of MACEs in a large longitudinally followed cohort. The prevalence of MetS in our population was nearly 40%, compared to 20%–30% in populations previously reported from Asia and worldwide.^4^, ^24^ This emphasizes the scale of this healthcare problem in Thailand. During a 12‐year follow‐up period, 8.2% of participants who had MetS at baseline developed a MACE and 11.2% died. The cause of death in MetS patients is mainly driven by cardiovascular death, with no significant difference in non‐CVD death. These numbers are higher than those found in a previous study by Laucevicius et al.^25^, which reported that 4.4% of MetS participants developed a CVE (and fatal CVEs comprised 6.5% of all CVEs), but the duration of follow‐up was shorter (3.8 ± 1.7 years). Most CVEs in that study and ours were caused by CAD rather than stroke. The strengths of this study were its prospective nature in a large community cohort, long duration of follow‐up, low out migration rate, and the verification and adjudication of each MACE and death by the expert review.

Arterial stiffness assessed by CAVI has been reported to be a surrogate marker of atherosclerosis and a predictor of future CVEs, independent of conventional risk factors.^16^ In our study, the prevalence of CAVI ≥ 9 was higher in participants with, than without, MetS. Having MetS itself carried a 5% higher risk of a MACE, but having CAVI ≥ 9 further increased this risk to 34%. The cut‐off of CAVI = 9 captured about 30% of the study group and added to the predictive value the diagnosis of MetS for MACEs.

In this study, we used the CAVI as a categorical rather than a continuous variables. A cut‐off of CAVI ≥ 9 is generally considered high and to represent the presence of atherosclerosis and as a predictor of CV risk.^22^, ^26^ This is consistent with previous studies that have shown CAVI ≥ 9 to be significantly increased in populations with metabolic CV risk factors, and to predict future CV risk in asymptomatic patients with type 2 DM.^27^, ^28^ However, there is still some discrepancy regarding the appropriate cut‐off value to use for discriminating CV risk. Sato et al.^16^ and Kubota et al.^29^ used a cut‐off of CAVI ≥ 10. Therefore, to determine the most appropriate CAVI values for prediction of MACEs, especially in MetS populations, further prospective studies are required.

Other risk factors, such as older age, male sex, are also reported to be associated with higher CAVI.^30^, ^31^ This might explain why participants in our study who had higher CAVIs were found to have an increased incidence of MACEs and ACD. Almost half of our participants had a history of cigarette use, and smoking is known to influence arterial stiffness.^32^ However, in the current study smoking was not significantly associated with MACEs. Furthermore, hypertension, DM, and dyslipidemia were also significantly associated with MACEs, particularly for DM and hypertension in which their effects were much higher than effect of MetS, including them into the same model could dilute effect and resulted in non‐significant effect of MetS. Most participants (especially in the MetS group) were under treatment with lipid lowering agents which resulted in their having lower total cholesterol and LDL‐C, and no association with MACEs.

In summary, in patients with MetS, CAVI was an additional risk predictor of future MACEs, independent of traditional coronary risk factors. CAVI was a potentially valuable non‐invasive test which identified high‐risk MetS patients who were likely to benefit from intensive therapeutic approaches.

Several limitations of the study need to be mentioned. The study population was enrolled only from among EGAT employees and were mostly middle or older age. Thus, the results may not be generalizable to younger populations. The definition of MetS was defined based on the baseline characteristics on the day of enrollment, and there is a possibility that some participants may have switched over during the follow‐up period. Such switches may have influenced the long‐term clinical outcomes. But since developing MetS was more likely than resolving the syndrome, this influence would tend to underestimate the true differences. Finally, even though this was a cross‐sectional, prospective cohort study, it could not prove causality between arterial stiffness and MACEs.

## 5 CONCLUSION

MetS is an important problem that is associated with MACEs and premature death. Arterial stiffness of a person can be assessed non‐invasively and semi‐quantitatively represented by CAVI, and this index can be used as a screening tool. CAVI may improve prediction of future MACEs, and also used to motivate MetS patients to modify their life‐style for CV disease prevention.

## CONFLICT OF INTEREST

None of the authors reported any conflicts of interest.

## Data Availability

The data that support the findings of this study are available on request from the corresponding author. The data are not publicly available due to privacy or ethical restrictions.
